# Arp’s Medical Reminiscences

**Published:** 1898-01

**Authors:** Bill Arp

**Affiliations:** Cartersville, Ga.


### ARP'S MEDICAL REMINISCENCES.

CARTERSVILLE, GA., December 10, 1897.

The *post-bellum* doctors of medicine can hardly realize the advance made in their profession in the last fifty or sixty years. We old men go back in memory to the ignorant simplicity of the doctors who ministered to us in youth and are amazed that we lived at all. But a kind Providence seems to have fortified us not only against the ills and accidents of life, but against the ignorance and blunders of the average country doctor. When I was a boy there were four physicians in our community. Two were allopaths, one was a steam doctor, and the other a water doctor. The last named had the largest practice, and bladders were emptied from all over the surrounding country that he might inspect their contents. Next there was the steam doctor, who had his patrons, and he got the dirt off of them and opened the pores, and effected some cures thereby.

But the allopaths held the fort among the better class. They had a few bottles on their shelves containing senna, camphor, salts, blue mass, jalap, lobelia, castor-oil, sweet-oil, and copaiba. These they administered freely, especially the castor-oil, to the negroes. They bled us fore and aft to reduce us, and then fed us on soup and gruel to form new bloods and build us up again. There was hardly any distinction in fevers. A fever was a fever, and there was the same treatment for all, and water was prohibited in all. I remember well when I would have given a world full of gold for a tumblerful. There were no drug stores, no pharmacy, no written prescriptions, no specialists for eye or ear or teeth. The village doctor was the dentist—and merciful heavens! what a horrible looking thing was the tool he used. It was modeled after a pair of those iron dogs that are used to turn a log in a sawmill. I have seen him hitch it on to a big negro's jaw-tooth and drag him out of his chair, and then break off a scale of the jaw-bone with it. But it always came.

These doctors used no antiseptics. They had never heard of morphine or quinine or bromide or vaseline or glycerin. If a patient was fated to recover he recovered. If not he died and died hard, for they wouldn't allow him to die easy. When they cut a man's leg off he had to grin and bear it. I was called on once to hold a man's leg, and when the saw cut through the bone and the weight of the severed limb came upon me I fell and fainted to the floor, for it made me awful sick—blood always does. Dr. Jim Alexander remembers that, for it was the leg of peg-leg Evans, and he knew him and was present. For some years Dr. Hall was the leading physician—a large, round, oily man of two hundred and fifty pounds. He rode a large, round, oily horse. Doctors always rode horseback then to their country patients, and Dr. Hall rode slow. He mounted and dismounted from a horse-block that was near his door, and constrained his country friends to provide a similar horse-block if they did not already have one for their women folks.

The doctor had such a vast corporosity that we boys, who early began to wonder where the babies came from, were easily persuaded that Dr. Hall had them in his paunch and distributed them around among the women who wanted them. Our old black mammy told us that.

But by and by this good old doctor died and we children missed him, for he gave us licorice root as we passed by his office and sometimes let us peep at the skeleton that hung in a long box in the corner.

Then came Dr. Wildman from New York, who was up to date in surgery and could cut the stone from the bladder and straighten cross-eyes and mend hare-lips, and remove cataracts, etc. He electrified and astounded the country—for he had an electric machine, a large, solid glass wheel that turned between two pads and generated electricity. Sometimes we boys and girls would form a circle and join hands and be shocked for the fun of it; for when the current was strong we couldn't let go and would squeeze the girls' hands harder and harder as they screamed. Sometimes a countryman would venture in and not daring to take hold himself was persuaded to hold up the forepaw of his dog and then they would both be knocked down together.

But by-and-by Wildman moved to Columbus and from there to Savannah to study yellow fever. The fever soon studied him and he died.

Then young Dr. Gordon, who married your Dr. Alexander's sister, and was a man of brains and ambition, resolved to investigate the fever, and like Wildman he took the pestilence and died, and the fever still stalks at noonday and at night, the terror and the dread of human kind.

Bill Arp.

---

### Vomiting in Pregnancy.

Page (*The Medical Times*, October, 1897) says of vomiting in pregnancy that "the first indication of nausea or lack of appetite, not to say vomiting, should warn a prospective mother that she is getting a little ahead of her stomach, so to say; and then all she has to do is simply to sip a few swallows of moderately hot water occasionally during the day, fasting, to make short and easy work of restoring the balance to the system. I have never known a single case of fuilure when this plan has been intelligently carried out. It may be necessary to skip only a couple of meals, or it may be necessary to fast two or three days. Wait until the patient is again really hungry before giving food. Meanwhile it will be necessary to give from a quart to three pints of soft warm water daily to fulfill the requirements of the system." The writer believes that many cases have terminated fatally in which life might have been saved by the adoption of this simple plan of treatment.—*Med. News.*

